# Meta-analysis of the impact of human papillomavirus (HPV) on cancer risk and overall survival in head and neck squamous cell carcinomas (HNSCC)

**DOI:** 10.1186/1758-3284-2-15

**Published:** 2010-06-29

**Authors:** Farshid Dayyani, Carol J Etzel, Mei Liu, Chung-Han Ho, Scott M Lippman, Anne S Tsao

**Affiliations:** 1 Department of Thoracic/Head & Neck Medical Oncology, University of Texas MD Anderson Cancer Center, Houston, 77030, USA; 2 Division of Cancer Medicine, University of Texas MD Anderson Cancer Center, Houston, 77030, USA; 3 Department of Epidemiology, University of Texas MD Anderson Cancer Center, Houston, 77030, USA

## Abstract

**Background:**

HPV is important in a subset of HNSCC. Our meta-analysis determined the clinical characteristics of HPV-related HNSCC.

**Method:**

Pubmed search terms "HPV" and "HNSCC" were used to identify 34 studies since 1980. We obtained pooled adjusted odds ratio (OR) or hazard ratio (HR) using random or fixed-effects model and compared OS depicted in forest plot.

**Results:**

A total of 5681 patients were included. The prevalence of HPV+ tumors was 22%, with 86.7% of HPV16+ genotype. The OR for HNSCC in HPV16+ patients was 4.44 (95% CI = 2.87-6.02). HPV status was associated with p16 expression (adj OR = 3.00; 0.90-9.70), and HPV+ tumors were less likely to harbor p53 mutations (adj OR = 0.21; 0.04-0.38). The HR for death in HPV+ patients was 0.42 (0.27-0.57). HPV+ HNSCC also had a better response to therapy.

**Conclusion:**

HPV+ HNSCC are established as a separate biologic entity. Prospective trials are needed to establish the optimal therapy for HPV+ HNSCC.

## Introduction

Squamous cell carcinoma of the head and neck (HNSCC) has an estimated incidence of 35,310 new cases in 2008 in the United States, with an expected 7590 deaths due to these cancers. The male to female ratio is approximately 2:1[1]. A recent analysis of the Surveillance, Epidemiology and End Results (SEER) database showed in younger U.S. populations (ages 20-44 years) that while there has been an increase in incidence of tonsillar squamous cell carcinomas from 1973 to 2001, the incidence of squamous cell carcinomas in all other oral and pharyngeal sites remained constant or decreased[2]. A similar rise in the incidence of tonsillar squamous cell carcinomas from 1970-2002 has been shown in Sweden and has been associated with presence of human papillomavirus (HPV)[3]. For more than 30 years, certain genotypes of "high-risk" HPVs have been known to be involved in the pathogenesis of cervical cancers[4]. Recent studies have implicated high-risk HPV as a risk factor for HNSCC, independent of the traditional risk factors, which include tobacco abuse and ethanol consumption[5-7]. While high-risk HPV related HNSCC appear to be associated with certain sexual behaviors, such as oral sex and increasing numbers of sexual partners, there is a lack of association with smoking and drinking[5,8]. Clinically, high-risk-HPV related HNSCC tend to present with lymph node positive disease and originate from the oropharynx, while histologically these tumors are usually high-grade and can be described as exhibiting a basaloid morphology [6,9,10]. On a molecular level, the HPV oncoproteins E6 and E7 are implied in tumorigenesis and are known to induce degradation of the tumor suppressors p53 and pRB, respectively[11,12]. Another hallmark of HPV related HNSCC is lack of p53 mutations[13] and p16 protein overexpression[14-16], a result of loss of transcriptional repression which occurs as a response to upstream signals during early tumorigenesis[17]. Studies suggest that p16 immunohistochemical expression can function as a surrogate marker to predict treatment outcome in HNSCC[14,18].

Recent emerging data indicates that HPV related HNSCC define a unique population of patients with distinct biology that likely should be treated separately from non-HPV related HNSCC[19]. The aims of this meta-analysis are to consolidate the available data, improve understanding of the biology of HPV related HNSCC, and thereby identify goals for future research and trial design in HNSCC.

## Methods

### Article selection

To identify articles, we searched the Pubmed database, combining permutations of the terms ["human papillomavirus" or "HPV] and ["squamous cell carcinoma" or "cancer"] and ["oropharyngeal" or "oropharynx" or "head and neck" or "tonsil"]. Additional articles from reference lists of retrieved articles were reviewed and included as deemed appropriate. Only articles which solely or separately reported on oropharyngeal cancers, were published from 1980-2008 in English language, and reported HPV prevalence were considered for inclusion. A total of 34 articles met our inclusion criteria for this meta-analysis.

### Statistical Analyses

The presence of study heterogeneity was evaluated using the Chi-squared based Q test. In order to quantify the extent (estimated as a percent ranging from 0% to 100%) of any existing heterogeneity in the meta-analyses, the I2 index was used. Pooled adjusted odds ratios (ORs) for risk or hazard ratios (HRs) for overall survival were obtained using a fixed-effects model (or random-effects model if needed due to among-study heterogeneity). If adjusted ORs or HRs were not available, then we used the same methods as above to pool unadjusted point estimates of risk and survival. Potential sources of publication bias were detected using a funnel plot which is simple scatter plots of the effect of interest versus study size. An asymmetrical funnel plot is indicative of potential publication bias. If necessary, we omitted studies prior to the final meta-analytic estimate that were identified as influential (those outside the allowable bounds based on the presumption of absence of both heterogeneity and publication bias) in the funnel plot. A forest plot was constructed to visually compare all included studies with the final pooled statistic. When possible, we also performed the above meta-analyses for the subset of oropharyngeal cancers. All analyses were completed using STATA 10.0.

## Results

### HPV Prevalence

A total of 5681 patients in 34 studies were included. To detect HPV DNA in tumor biopsies, 33 studies used PCR, and one study used fluorescence in situ hybridization (FISH). We established a prevalence of HPV among all head and neck squamous cell carcinoma (HNSCC) patients of 21.95% (95% CI = 0.21-0.23). The most prevalent HPV genotype was HPV-16, representing 86.69% (95% CI = 0.85-0.89) of all HPV positive tumors (Table 1 and 2). The other two most commonly detected genotypes were HPV-18 and -33 (data not shown). In the publications which included information on the subgroup of oropharyngeal cancers, the prevalence of HPV was 41% (379 of 925 patients; 95% CI = 0.38-0.44).

**Table 1:** HPV Prevalence in 34 trials (N = 5681)

Title	Patients (n)	No. of HPV positive (n)	Prevalence HPV %	No. of HPV16 (n)	Prevalence HPV16 in HPV (%)
Mork rtl, et al. 2001	292	88	30.14%	35	39.77%
D'Souza, et al. 2007	100	40	40.00%	35	87.50%
Gillison, et al. 2000	253	62	24.51%	56	90.32%
Herrero, et al. 2003	1670	56	3.35%	21	37.50%
Smith, et al. 2004	201	46	22.89%	39	84.78%
Braakhuis, et al. 2004	143	24	16.78%	24	100.00%
Fakhry, et al. 2008	96	38	39.58%	36	94.74%
Teman, et al. 2008	105	12	11.43%	0	0.00%
Weinberger, et al. 2006	77	47	61.04%	47	100.00%
Licitra, et al. 2006	90	17	18.89%	17	100.00%
Worden, et al. 2008	66	27	40.91%	27	100.00%
Shindoh, et al. 1995	77	24	31.17%	24	100.00%
Lindel, et al. 2001	99	14	14.14%	11	78.57%
Klussmann, et al. 2001	98	25	25.51%	21	84.00%
Perrone, et al. 2007	77	16	20.78%	16	100.00%
Kutler, et al. 2003	25	21	84.00%	19	90.48%
Smith, et al. 2004	193	38	19.69%	33	86.84%
Baez, et al. 2004	118	52	44.07%	52	100.00%
Dahlgren, et al. 2004	110	12	10.91%	9	75.00%
Hoffmann, et al. 2004	73	28	38.36%	28	100.00%
Slebos, et al. 2006	36	8	22.22%	8	100.00%
Hammarstedt, et al. 2006	203	99	48.77%	86	86.87%
Ragin, et al. 2006	125	30	24.00%	30	100.00%
Reimers, et al. 2007	106	30	28.30%	29	96.67%
Furniss, et al. 2007	486	145	29.84%	145	100.00%
Smith, et al. 2008	301	81	26.91%	77	95.06%
Pintos, et al. 2008	72	14	19.44%	13	92.86%
Charfi, et al. 2008	52	32	61.54%	27	84.38%
Westra, et al. 2008	89	12	13.48%	12	100.00%
Hafkamp, et al. 2008	81	33	40.74%	33	100.00%
Friesland, et al. 2001	34	14	41.18%	14	100.00%
De Petrini, et al. 2006	47	20	42.55%	20	100.00%
Wittekindt, et al. 2005	34	18	52.94%	16	88.89%
Strome, et al. 2002	52	24	46.15%	21	87.50%

	**5681**	**1247**	**21.95%**	**1081**	**86.69%**

**Table 2:** Selected characteristics of the 34 studies

		N	%
**Confounding factors**	Age	22	64.71%
	Gender	24	70.59%
	Smoking	15	44.12%
	Alcohol	15	44.12%

**Study type**	Case-control	6	17.65%
	Nested Case Control	1	2.94%
	Multi-Center Case Control	1	2.94%
	Case Series	3	8.82%
	Retrospective	20	58.82%
	Prospective	3	8.82%

**Sample size**	< 50	5	14.71%
**(No. of patients)**	50-99	14	41.18%
	100-149	7	20.59%
	>150	8	23.53%

**Prevalence HPV**	< 25%	14	41.18%
	25%-50%	14	41.18%
	50%-75%	5	14.71%
	>75%	1	2.94%

**Prevalence HPV-16**	< 25%	16	47.06%
	25%-50%	13	38.24%
	50%-75%	4	11.76%
	>75%	1	2.94%

**Method of Detection**	PCR	33	97.06%
	FISH	1	2.94%

**Country**	USA	14	41.18%
	Canada	1	2.94%
	Puerto Rico	1	2.94%
	France	2	5.88%
	Germany	4	11.76%
	Netherlands	2	5.88%
	Italy	3	8.82%
	Switzerland	1	2.94%
	Norway, Finland, Sweden	1	2.94%
	Sweden	3	8.82%
	Japan	1	2.94%
	International	1	^2.94%^

### HPV as Risk Factor for HNSCC

**Figure 1:**
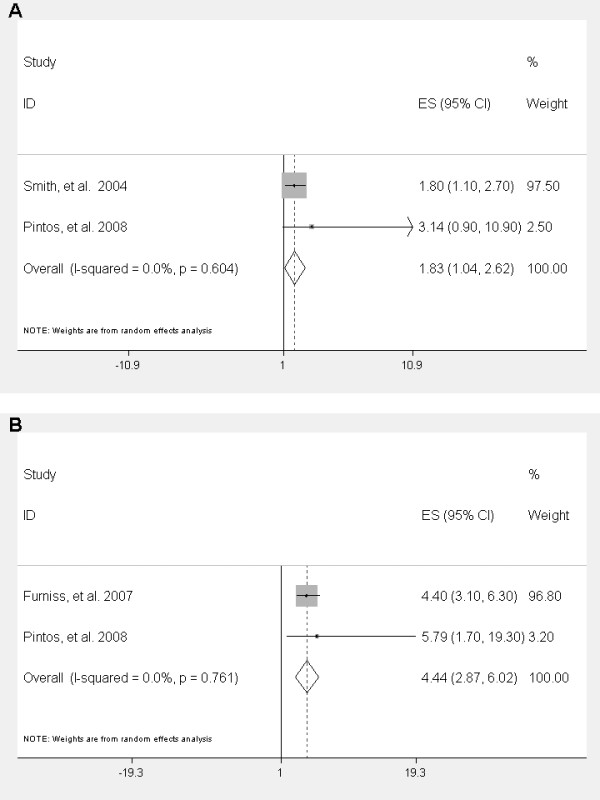
**Meta-analyses for adjusted odds ratio (OR) for HPV as risk factor for HNSCC Risk for having HNSCC is increased with all types of high-risk HPV reported (A) and risk is even more pronounced in cases with HPV-16 infection versus HPV negative controls (B)**.

Overall, positive HPV status (any high-risk genotype) conferred an increased risk for HNSCC (adjusted OR = 1.83; 95% CI = 1.04-2.62; p < 0.0001) as seen in Figure 1A. Risk for HNSCC among HPV-16 positive patients was 4.44 times (95% CI = 2.87-6.02; p < 0.0001) that of HPV-16 negative patients (Figure 1B). As a validation control for our meta-analysis we assessed the OR of two known risk factors for HNSCC, tobacco and alcohol consumption[20,21]. We confirmed that heavy smokers and alcohol drinkers are at increased risk for HNSCC (smoking OR = 3.53; 95% CI = 2.69-4.64, p < 0.0001, and alcohol consumption OR = 4.04; 95% CI = 3.20-5.09, p < 0.0001). Table 3 shows the association of HPV status with prognostic factors for HNSCC (age, gender, and tumor stage) and known risk factors for HNSCC (ethanol and tobacco consumption)

**Table 3:** Association of HPV status with gender, age, tumor stage, alcohol and tobacco consumption

Demographic	# Studies	HPV status	Smoking status	N	%
Tobacco	17	HPV+	Ever	1068	55.9
			Never	948	44.1
		HPV-	Ever	2179	74.5
			Never	677	25.5
Ethanol	16	HPV+	Ever	1548	66.8
			Never	768	33.2
		HPV-	Ever	2186	75.4
			Never	713	24.6
Stage	10*	HPV+	I-II	78	26.7
			III-IV	214	73.3
		HPV-	I-II	259	42.8
			III-IV	346	57.2
LN Stage	6	HPV+	N0	45	33.5
			N1-N3	123	66.5
		HPV-	N0	204	45.9
			N1-N3	240	54.1
Gender	18	HPV+	M	1525	67.8
			F	724	32.2
		HPV-	M	2010	66.1
			F	1033	33.9
Median Age	5**	HPV+	55 years		
		HPV-	60 years		
					

HPV: human papillomavirus; M: male; F: female; LN, lymph node; * 2 studies compared stage I-III vs. stage IV; ** 5 studies reported the median age of the subjects.

### HPV and Survival

**Figure 2:**
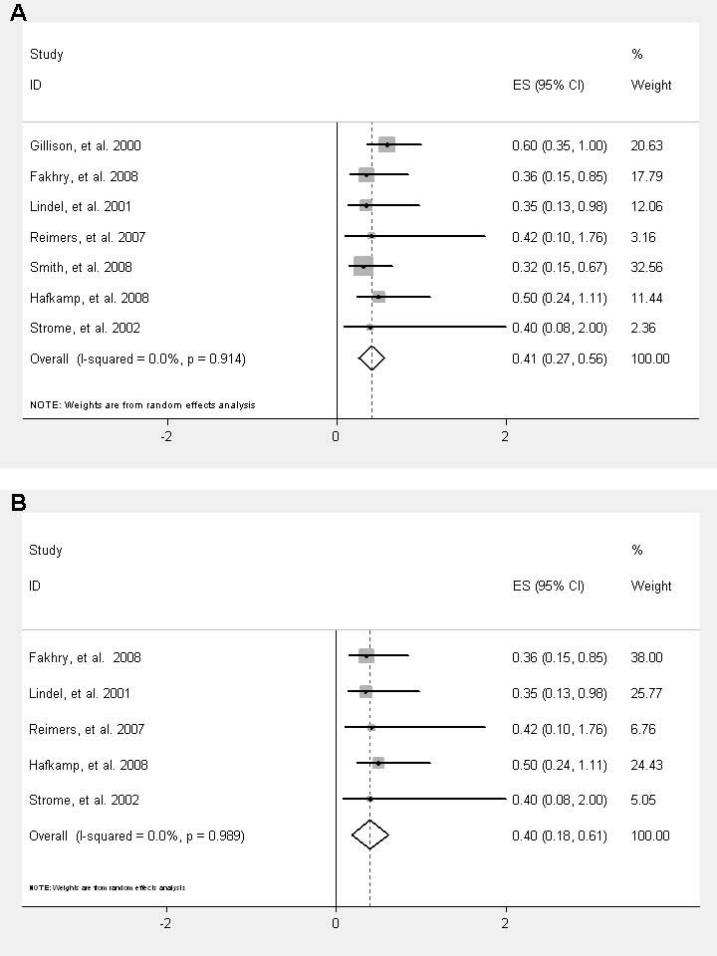
**Overall survival of HPV positive patients compared to HPV negative patients in unselected HNSCC patients (A) and in the subgroup of patients with oropharyngeal cancers (B)**.

Survival was improved in HPV positive patients compared to HPV negative patients (HR = 0.42; 95% CI = 0.27-0.56, p < 0.0001) (Figure 2A). The survival benefit was similar in HPV-16 positive patients (adjusted HR = 0.41; 95% CI = 0.21-0.61, p < 0.0001). The improved survival in HPV positive patients was even more pronounced in the subgroup of oropharyngeal cancers (adjusted HR = 0.40; CI = 0.18-0.61, p < 0.0001) (Figure 2B).

### HPV and Treatment Response

A total of ten publications included treatment information, but only two reported statistical information on treatment response to radiotherapy and two reported efficacy results to chemotherapy/radiotherapy (Table 4). HPV positive HNSCC have a better response to radiotherapy compared to HPV negative HNSCC (non-adjusted OR = 4.07; 95% CI = 1.48-11.18, p = 0.006). Similarly, patients who are positive for HPV have an improved response to concurrent chemo-radiation (non-adjusted OR = 2.87; 95% CI = 1.29-6.41, p = 0.01).

**Table 4:** Treatment type and country of origin of studies that reported treatment information

Author, Year	Type of Treatment	Country
Fakhry, et al. 2008	CTX followed by Chemo-XRT	United States
Weinberger, et al. 2006	XRT ± surgery	United States
Worden, et al. 2008	CTX followed by Chemo-XRT or XRT/surgery	United States
Lindel, et al. 2001	XRT	Switzerland
Dahlgren, et al. 2004	XRT	Sweden
Smith, et al. 2008	XRT ± surgery	United States
Hafkamp, et al. 2008	CTX ± XRT ± surgery	Netherlands
Friesland, et al. 2001	XRT	Sweden

CTX: Chemotherapy; XRT: Radiotherapy; Chemo-XRT: concurrent chemo-radiation

### Correlation with p16 and p53

Several studies reported p16 immunohistochemical expression in HPV positive HNSCC[22-29]. We found that HPV status was associated with p16 expression (adjusted OR = 3.00; 95% CI = 0.90-9.70, p = 0.18), and HPV positive tumors were less likely to harbor p53 mutations (adjusted OR = 0.21; 95% CI = 0.04-0.38, p = 0.015) (Table 5). Confirming the notion that p16 expression might be used as a surrogate for HPV status in HNSCC, we show that p16 positive HNSCC have reduced hazard ratio for death (adjusted HR = 0.22; 95% CI = 0.02-0.41, p = 0.028).

**Table 5:** Meta-analyses of association between p53 mutations, p16 and HPV

		Before outlier exclusion		After outlier exclusion	
Outcome	Marker	No. of Studies	Adjusted OR (95% CI)	No. of Studies	Adjusted OR (95% CI)
HPV positive	p53 (mutated)	4	0.21(0.04-0.38)	4	0.21(0.04-0.38)
					P = 0.015
HPV positive	p16	2	5.02(1.08-8.97)	1	3.00(0.90-9.70)
	IHC				p = 0.181
					

## Discussion

We have conducted a literature review and large meta-analysis in HPV related HNSCC to determine the prevalence of the disease and assess the ramifications on survival and associated molecular biomarkers. Given prior published reports of a higher prevalence of HPV in carcinomas of the oropharynx, we concentrated this study on oropharyngeal carcinomas to enrich and focus the meta-analysis. Therefore, the number of studies included in our review is smaller than other previously reported meta-analyses with different inclusion criteria[30,31]. Several other meta-analyses on HPV-related HNSCC have been published before[30-32], but to our knowledge, none of the prior reports have described within the same report, the impact of HPV on survival, response to therapy, as well as the association with molecular markers such as p16. In addition, our report includes recently published prospective studies that were not available at the time of publication of previous meta-analyses. Our data reports the prevalence of HPV-related HNSCC as 21.95% and is in accordance with other recently published reports which estimate the prevalence of HPV at 20-26% [25,30-32]. As expected, we also confirmed a higher prevalence of HPV-related tumors in the oropharynx (40.97%) in agreement with prior studies[31,33]. Virtually all of the studies included used PCR to detect HPV DNA, a method with slightly higher sensitivity than in situ hybridization[31]. The higher sensitivity of the detection method used suggests that the herein reported prevalence of HPV associated HNSCC is more likely to reflect the actual prevalence of HPV as compared to studies which use less sensitive methods, although given the paucity of data regarding the exact specificity of PCR in this setting does not enable a precise account of the number of false-positive patients included in these studies. The relatively wide range of the prevalence of HPV in HNSCC in the published literature confirms that in addition to geographical differences, the method of HPV detection is critical for accurate assessment of the HPV status.

In the current study we confirmed the role of p16 immunohistochemical expression as a surrogate marker for HPV infection in HNSCC, which is not only positively associated with HPV infection but also predicts for an improved survival outcome[14,28,29]. The HPV oncoprotein E7 is known to induce degradation of pRb, which in turn leads to upregulation of p16[34]. Therefore, overexpression of p16 is considered to be one of the molecular hallmarks of HPV positive HNSCC[4,23,35,36] and has recently been shown to identify HPV related oropharyngeal cancers with favorable outcomes[16]. In a recent prospective trial, an analysis of tumor tissue (n = 323) collected from RTOG 0129 using HPV in situ hybridization (ISH) and p16 immunohistochemistry (IHC) demonstrated that p16 correlated well with HPV ISH with 96% of HPV positive tumors also expressing p16[19]. An additional 19% (n = 22) were p16 positive but HPV negative. Both HPV and p16 positive status predicted for an improved survival. This finding of p16 IHC positive tumors identifying more patients than HPV ISH was also seen in an additional study called HeadStart[37]. This trial compared HPV ISH and p16 IHC on tumor specimens from two arms of therapy, cisplatin-radiation versus cisplatin-tirapazamine-radiation, and suggested that p16 IHC may identify a higher percentage of HPV positive tumors than the HPV ISH assay. Given the debate regarding the best detection method for HPV[38], our data from the meta-analysis confirms that p16 IHC could be used in future in prospective trials as a reliable surrogate marker for HPV related HNSCC[19,37,39]. As the above two recent studies (RTOG and HeadStart) have only been reported in abstract form, we did not include them in our meta-analysis.

As HPV-related oropharyngeal cancers are reported to be rising in incidence[2,3], it is important to consider treating this disease as a unique entity. Our data in congruence with other studies indicate that HPV positive HNSCC have a distinct biology, and are more responsive to treatment with radiotherapy and chemotherapy. In addition to treatment response, we and others have shown an improved survival in patients with HPV positive HNSCC, with a 60% lower risk of death. Our survival results are slightly different from other reports; a recent meta-analysis reported a death hazard ratio of 0.85. This discrepancy can be explained by the addition of the recent prospective trials[10,14,40,41] in our analysis which were not included in prior reports. Our meta-analysis also confirms a better response to treatment in HPV positive HNSCC in accordance with recently published trials[10,14,39,41,42]. Recent reports have highlighted the importance of the negative influence of smoking on prognosis within HPV positive HNSCC[19,43], and future studies are required to examine this relationship in a prospective manner.

There are several limitations inherent to our methodology, which was based on literature review, not individual patient data. While the total number of patients included in our meta-analysis is large, for many specific associations the number of the studies that actually could be combined to provide information is relatively small. This is a limitation which unfortunately cannot be overcome without access to primary data from all included publications. Also, there is overlap of the same patient series included in multiple reports. We tried to account for this fact as much as possible by reviewing the author list and institution of each paper and including only the largest study if several were apparently from the same group. Finally, given the heterogeneity of the kinds of studies our meta-analysis was based on, it is difficult to combine conclusions drawn, e.g. assessing HPV as risk factor for HNSCC from case series, retrospective and prospective trials. There is no valid formulation to give adequate weight to the results of each kind of the studies mentioned. In spite of the limitations inherent to this type of a meta-analysis, our results are in keeping with published literature and our current understanding of the natural biology of HPV-related HNSCC. We also performed as a validation control a meta-analysis for the established HNSCC risk factors smoking and alcohol consumption. Our results confirmed prior published results [20,21].

For the future, there is a need for randomized prospective data to identify the optimal treatment for patients based on the tumor's HPV status and to also develop potential prevention programs. First, given the apparent improved response to current treatment modalities, patients with HPV-related HNSCC may be able to receive adequate curative intent therapy from a reduction in radiation or chemotherapy dose. These issues will require future clinical trial designs to either prospectively stratify patients by HPV status or to conduct trials which are dedicated to HPV positive HNSCC. In addition, it is necessary to determine the best method of detecting the HPV status and validate whether p16 overexpression should be utilized as a surrogate or in conjunction with direct HPV testing. Given the etiologic involvement of the HPV oncoproteins E6 and E7 during carcinogenesis of these tumors[44], it is also conceivable that intervention with anti-viral drugs may enhance sensitivity of these tumors to cytotoxic drugs, with the goal of reducing the required dose of chemotherapeutic agents and the associated side effects. Exploratory biomarker studies will be needed to identify prognostic and predictive markers in order to tailor specific therapies (such as biologic agents) to the biology of the patients' malignancy, resulting in better tolerability of treatment with improved outcomes.

The issue of preventing HPV associated HNSCC from occurring requires investigation as well. Two recently approved vaccines to prevent HPV-related cervical cancers[45,46] cover the two serotypes that are most prevalent in HPV-related HNSCC (HPV-16 and 18). In the context of the findings of our study, it is conceivable that extending vaccination to both sexes would prevent a significant number of future oropharyngeal cancers in both men and women. Also, the hypothesized benefits of HPV-vaccination would potentially include a reduced incidence of other HPV-related cancers (i.e. vulvar, vaginal, anal, and penile cancers)[47]. On the other hand, a recent study showed that vaccination of males on a population basis might not be cost-effective[48]. Even in women, controversy exists regarding the maximum age at which the HPV-vaccine should be given, and a recent simulation model suggested that vaccination beyond 30 years of age might also not be cost-effective[49]. Given all these uncertainties, it will therefore be of great importance to assess in prospective trials that include both sexes the efficacy of these vaccines to prevent HPV-related HNSCC.

## Conclusions

HPV-related HNSCC comprise about 25% of all HNSCC. They are predominantly tumors of the oropharynx, and exhibit a separate biologic behavior including improved response to (chemo)-radiation and survival compared to HPV-negative HNSCC. The optimal therapy for this subset of patients remains unclear, but the growing data supports the theory that this subset of patients should be treated differently than HPV negative patients. In addition to this avenue of clinical research, identifying peripheral surrogate biomarkers and possible screening and prevention methods is an important field of future investigation.

## Declaration of Competing Interests

The authors declare that they have no competing interests.

## Authors' contributions

FD and AST designed the study, acquired and interpreted the data, and wrote the manuscript. CJE, ML, and CHH acquired, analyzed, and interpreted the data, and contributed to manuscript writing. SML contributed to study design, data interpretation and manuscript writing. All authors have given final approval of the version to be published.
